# Combining Sparse Group Lasso and Linear Mixed Model Improves Power to Detect Genetic Variants Underlying Quantitative Traits

**DOI:** 10.3389/fgene.2019.00271

**Published:** 2019-04-10

**Authors:** Yingjie Guo, Chenxi Wu, Maozu Guo, Quan Zou, Xiaoyan Liu, Alon Keinan

**Affiliations:** ^1^School of Computer Science and Technology, Harbin Institute of Technology, Harbin, China; ^2^Department of Computational Biology, Cornell University, Ithaca, NY, United States; ^3^Department of Mathematics, Rutgers University, Piscataway, NJ, United States; ^4^School of Electrical and Information Engineering, Beijing University of Civil Engineering and Architecture, Beijing, China; ^5^Institute of Fundamental and Frontier Sciences, University of Electronic Science and Technology of China, Chengdu, China; ^6^Cornell Center for Comparative and Population Genomics, Center for Vertebrate Genomics, and Center for Enervating Neuroimmune Disease, Cornell University, Ithaca, NY, United States

**Keywords:** genome-wide association studies, single nucleotide polymorphisms, quantitative traits, linear mixed model, sparse group lasso

## Abstract

Genome-Wide association studies (GWAS), based on testing one single nucleotide polymorphism (SNP) at a time, have revolutionized our understanding of the genetics of complex traits. In GWAS, there is a need to consider confounding effects such as due to population structure, and take groups of SNPs into account simultaneously due to the “polygenic” attribute of complex quantitative traits. In this paper, we propose a new approach SGL-LMM that puts together sparse group lasso (SGL) and linear mixed model (LMM) for multivariate associations of quantitative traits. LMM, as has been often used in GWAS, controls for confounders, while SGL maintains sparsity of the underlying multivariate regression model. SGL-LMM first sets a fixed zero effect to learn the parameters of random effects using LMM, and then estimates fixed effects using SGL regularization. We present efficient algorithms for hyperparameter tuning and feature selection using stability selection. While controlling for confounders and constraining for sparse solutions, SGL-LMM also provides a natural framework for incorporating prior biological information into the group structure underlying the model. Results based on both simulated and real data show SGL-LMM outperforms previous approaches in terms of power to detect associations and accuracy of quantitative trait prediction.

## 1. Introduction

Quantitative traits are important in medicine, agriculture, and evolution but, until recently, few polymorphisms have been shown to be related in these traits. Genome-wide association studies (GWAS) is a statistical technique that has been used successfully in the identification of over 65,000 single-nucleotide polymorphisms (SNPs) that are connected to various traits or diseases (MacArthur et al., 2017). Typically, GWAS are carried out using single-locus models (i.e., testing for association between each SNP and a given phenotype independently using linear or logistic regression). However, according to the popular “polygenic theory” (Li et al., 2015b; Dudbridge, 2016), complex traits are often controlled by multiple SNPs collectively. Due to the need to eliminate multi-testing corrections that decrease statistical power, a better understanding of the underlying heritable genetic architecture of complex traits requires one to move beyond single-locus models to multivariate linear regression models that incorporate the joint effects of multiple SNPs explicitly (Ma et al., 2013).

Usually, the multi-locus GWAS are large p small n problems (i.e., the number of features (SNPs) far exceeds the number of samples, and one would expect only a small number of features are associated with the phenotype predictor). Therefore, as is customary for similar regression problems, it is necessary to regularize by demanding sparsity in the coefficients of the final model to prevent over-fitting and to maintain interpretability. The most popular regularizing penalty that serves this purpose is the lasso (i.e., least absolute shrinkage and selection operator) (Tibshirani, 1996), which is the L1 norm of the coefficients of features. Yang et al. (2012) fit sparse predictors for all genome-wide SNPs using stepwise, forward selection. Li et al. (2011) imposed a Laplace prior, which led to the Bayesian lasso. Arbet et al. (2017) developed a permutation-based, selection procedure to test the significance of lasso coefficients.

In GWAS, one expects the effective SNPs to be clustered in a few genes or pathways, hence, adding group structure by mandating sparsity on the group level is a good way to apply this prior knowledge that can potentially outperform the simple lasso. Yuan and Lin (2006) proposed using the group lasso for the linear regressions, which imposed a regularization penalty of the sum of the L2 norm on groups that guaranteed that few groups were selected. But if a group is selected, so are all the predictors in it.

The group lasso has already enjoyed much success in GWAS (Li et al., 2015a; Lim and Hastie, 2015). A caveat, however, is its assumption that either all SNPs in a group being associated or none of the SNPs in a group being associated. It is desirable to not only constrain sparsity between groups (only a few groups are associated), but also within groups; only a few SNPs in each active group are associated. Hence, we propose to employ a sparse group lasso (SGL), which is a regularization method aimed at achieving both between- and within-group sparsity simultaneously (Rao et al., 2013, 2016; Simon et al., 2013). The SGL has a L2 penalty that promotes the selection of only a subset of the groups and L1 penalty that promotes the selection of only a subset of the predictors within a group.

Another important factor in genetic association studies is the existence of confounding, which are indirect associations between markers and traits due to factors like population structure, family structure, and cryptic relatedness. Methods for correcting these confounding factors include EIGENSTRAT (Price et al., 2006), family-based association, genomic control, and linear mixed models (LMMs) (Fisher, 1919; Hoffman, 2013; Hoffman et al., 2014). Compared with other methods, LMMs provide more fine-grained control by modeling the contribution of these confounders as a random effect term. They are capable of capturing the cumulative effect of all types of confounding simultaneously without the need of prior knowledge on which confounding is present and without the need to estimate them individually. However, the time and space costs of LMM are high compared with simpler confounding models. Previous attempts to improve the performance of LMM includes Zhou and Stephens (2012) (EMMA), Kang et al. (2010) (EMMAX), Zhang et al. (2010) (P3D), Lippert et al. (2011) (FaST-LMM), and Li et al. (2017) (StepLMM). All of these methods are univariate models that are powerful in detecting few associations with large effect sizes.

Although joint modeling of multiple weak effects and correction for population structure have been tackled individually, few existing methods are capable of addressing them simultaneously. Segura et al. (2012) proposed a multi-locus, mixed model approach using stepwise forward selection. Rakitsch et al. (2012) and Papachristou et al. (2016) developed new association methods that combined LMM and lasso to enjoy the benefits of both methods.

**Figure 1:**
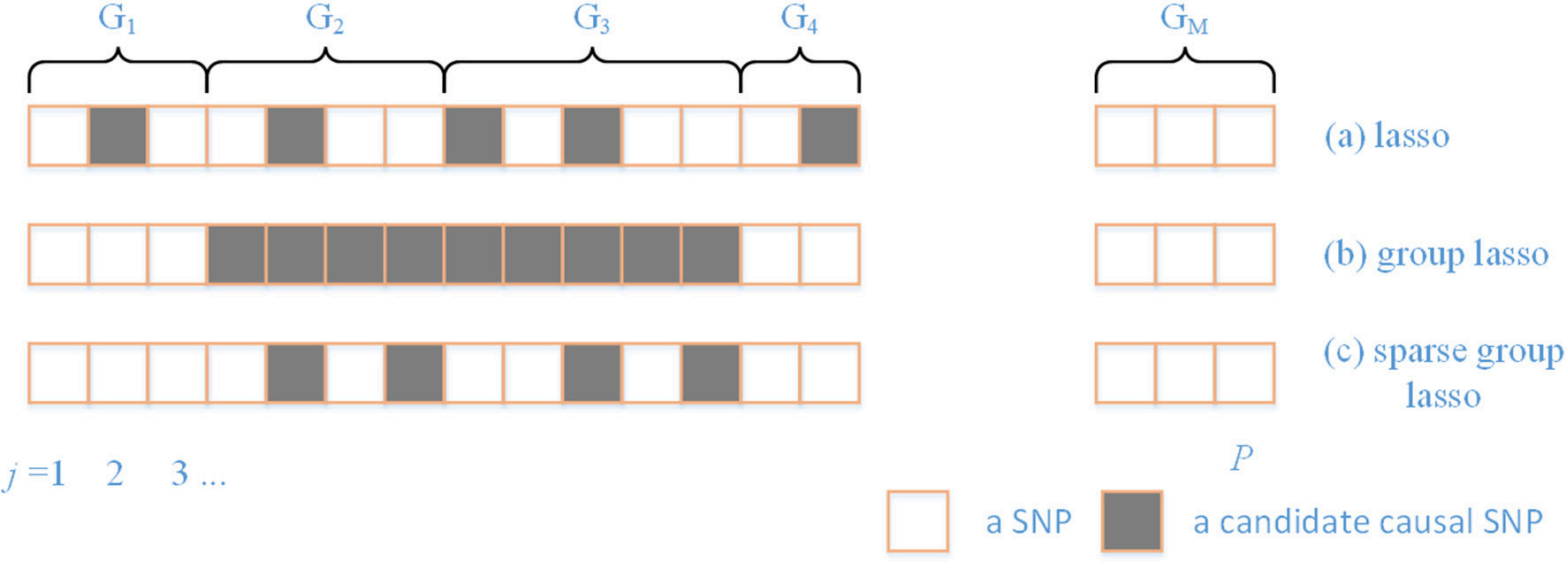
A comparison of different sparsity patterns that occur in the analysis of genome-wide association studies. SNPs belong to M genes. Association SNPs that influence the phenotype are represented by boxes that are shaded gray. **(a)** Shows a lasso sparse pattern. An example of the group sparse pattern is shown in **(b)**. In **(c)**, we show the pattern in which we are interested in this paper.

There are a variety of patterns that typically arise in regularization (Figure 1). Prior knowledge can be utilized by using the SGL, which maintains both between- and within-group sparsity. The relative strength between L1 and L2 norms can be used to represent prior knowledge on the comparative degrees of sparsity at the SNP and gene level. In particular, by varying the ratio between L1 and L2 norms, the approach includes both group lasso and lasso as special cases.

In this paper, we propose a novel analysis that not only combines multivariate analysis with population correction using Fast-LMM, but we also incorporate the group structure of the SNPs as biological priors. We use the gene as the group unit, and it is reasonable to assume that the model should be sparse not only on the SNP-level (only relatively few SNPs are involved), but on the gene level as well (those functional SNPs belong to relatively few genes). Experiments on semi-empirical data showed that the combination of sparse group lasso and a linear mixed model yielded better power to identify marker associations in a large range of settings, and application to real datasets have verified that SGL-LMM generated a sparse solution with accurate prediction of phenotypes and interpretable detection of marker associations.

## 2. Materials and Methods

### 2.1. Method

We used a linear mixed model to model the genetic effects on the phenotypes. More precisely, we modeled the phenotype as a sum of three terms: a fixed effect determined by the association SNPs, a random confounding effect due to population structure, and an i.i.d. noise as follows:

(1) $$\begin{array}{l} \text{y}=\text{X}\beta +{\text{y}}_{pop}+\phi \end{array}$$

where *y* is a vector of observed phenotypes of size *m* × 1 for *m* samples, *X* is a *m* × *q* matrix that consists of SNPs and other (e.g., environmental, familial etc.) variables of the *m* samples, ***y***_*pop*_ is a *m* × 1 random matrix with distribution $N\left(0,\sigma_{g}^{2}K\right)$ where ***K*** is an *m* by *m* matrix called realized relationship matrix(RRM) that captures the overall genetic similarity between all pairs of samples, and $\phi \sim N\left(0,\sigma_{e}^{2}I\right)$.

To make a prediction on ***y***, one only needs **β** and $\delta =\frac{\sigma_{e}^{2}}{\sigma_{g}^{2}}$. Following FAST-LMM, our overall strategy for estimating the parameters **β** and δ goes as follows:

1. Set **β** = 0, find the optimal δ.
2. Use the δ from the first step to estimate **β**, regularizing by using SGL.

Now we describe each of the two steps in more detail.

#### 2.1.1. Estimate of δ

To calculate δ we use an approach similar to Fast-LMM. Because **β** was set to 0, we have:

(2) $$\begin{array}{l} \text{y}\sim N\left(0,\sigma_{g}^{2}\left(\text{K}+\delta \text{I}\right)\right) \end{array}$$

Hence the log likelihood for a given ***y*** is

(3) $$\begin{array}{l} LL(\delta ,\sigma_{g}^{2})=\log N(0,\sigma_{g}^{2}(K+\delta I))\\ \;=-\frac{1}{2}(m\log(2\pi \sigma_{g}^{2})+\log(\det(K+\delta I)\\ \;+\frac{1}{\sigma_{g}^{2}}y^{T}{\left(K+\delta I\right)}^{-1}y) \end{array}$$

Diagonalize ***K*** into ***K*** = ***USU***^*T*^ where ***U*** is orthogonal and *S* is diagonal, and we have:

(4) $$\begin{array}{l} LL(\delta ,\sigma_{g}^{2})=-\frac{1}{2}(m\log(2\pi \sigma_{g}^{2})+\log(\det(S+\delta I)\\ +\frac{1}{\sigma_{g}^{2}}{\left(U^{T}y\right)}^{T}{\left(S+\delta I\right)}^{-1}(U^{T}y)) \end{array}$$

Substitute $\sigma_{g}^{2}$ with the optimal value:

(5) $$\begin{array}{l} \hat{\sigma_{g}^{2}}=\frac{{\left(U^{T}y\right)}^{T}{\left(S+\delta I\right)}^{-1}\left(U^{T}y\right)}{m} \end{array}$$

we have:

(6) $$\begin{array}{l} LL\left(\delta \right)=-\frac{1}{2}\left(\log\left(\det\left(\text{S}+\delta \text{I}\right)\right)+m\log\frac{{\left({\text{U}}^{T}\text{y}\right)}^{T}{\left(\text{S}+\delta \text{I}\right)}^{-1}\left({\text{U}}^{T}\text{y}\right)}{m}\right)\\ +C \end{array}$$

Where *C* does not depend on δ. The optimal δ can then be calculated from above as a one dimensional optimization problem:

(7) $$\begin{array}{l} \hat{\delta }=\arg\min\left(\log\left(\det\left(\text{S}+\delta \text{I}\right)\right)+m\log\frac{{\left({\text{U}}^{T}\text{y}\right)}^{T}{\left(\text{S}+\delta \text{I}\right)}^{-1}\left({\text{U}}^{T}\text{y}\right)}{m}\right) \end{array}$$

#### 2.1.2. Estimate of β

In this subsection, we describe the estimation for **β** based on model described by Equation (1), then, in the next subsection, we introduce the SGL regularization.

Equation (1) implies that:

(8) $$\begin{array}{l} y\sim N\left(X\beta ,\sigma_{g}^{2}\left(K+\delta I\right)\right) \end{array}$$

Hence, using the diagonalization we see that, after δ and $\sigma_{g}^{2}$ have been estimated in the previous subsection, the log-likelihood becomes:

(9) $$\begin{array}{l} LL(\beta )=\log N\left(X\beta ,\hat{\sigma_{g}^{2}}\left(K+\hat{\delta }I\right)\right)\\ \;=-\frac{m}{2}\log\left(2\pi \hat{\sigma_{g}^{2}}\right)-\frac{1}{2}\log(\det\left(S+\hat{\delta }I\right)\\ \;-\frac{1}{2\hat{\sigma_{g}^{2}}}\left(U^{T}\left(y-X\beta \right){)}^{T}{\left(S+\hat{\delta }I\right)}^{-1}\left(U^{T}(y-X\beta \right)\right)\\ \;=-\frac{1}{2\hat{\sigma_{g}^{2}}}\left(U^{T}\left(y-X\beta \right){)}^{T}{\left(S+\hat{\delta }I\right)}^{-1}(U^{T}\left(y-X\beta \right)\right)+C \end{array}$$

Let $S_{\hat{\delta }}$ be the non-negative diagonal matrix defined by $S_{\hat{\delta }}^{-2}=S+\hat{\delta }I$, or, more concretely, ${\left(S_{\hat{\delta }}\right)}_{ii}={\left(S_{ii}+\hat{\delta }\right)}^{-1/2}$, then the MLE of **β** is

(10) $$\begin{array}{l} \hat{\beta }=\arg\min{\left({\text{U}}^{T}\left(\text{y}-\text{X}\beta \right)\right)}^{T}{\left(\text{S}+\hat{\delta }\text{I}\right)}^{-1}\left({\text{U}}^{T}\left(\text{y}-\text{X}\beta \right)\right)\\ =\arg\min{\left({\text{S}}_{\hat{\delta }}{\text{U}}^{T}\text{y}-{\text{S}}_{\hat{\delta }}{\text{U}}^{T}\text{X}\beta \right)}^{T}\left({\text{S}}_{\hat{\delta }}{\text{U}}^{T}\text{y}-{\text{S}}_{\hat{\delta }}{\text{U}}^{T}\text{X}\beta \right)\\ =\arg\min||S_{\hat{\delta }}U^{T}y-S_{\hat{\delta }}U^{T}X\beta |{|}_{2}^{2} \end{array}$$

Here || · ||_2_ is the *L*^2^ norm. $S_{\hat{\delta }}U^{T}y$ and $S_{\hat{\delta }}U^{T}X$ are obtained from ***y*** and ***X*** by a rotation and a scaling, and to simplify notations we denote them as $\tilde{y}$ and $\tilde{X}$, respectively.

#### 2.1.3. Sparse Group Lasso

To maintain sparsity in the estimated **β**, we need to add a regularizer to Equation (10). We used the SGL regularizer: let G be a family of possibly overlapping groups of components in **β**, for each group G∈G, let **β**_*G*_ be the vector that consists of these components, let λ > 1 and 0 ≤ α ≤ 1, then the regularized optimization problem becomes:

(11) $$\begin{array}{l} {\hat{\beta }}_{reg}=\arg\min||{\text{S}}_{\delta }{\text{U}}^{T}\text{y}-{\text{S}}_{\delta }{\text{U}}^{T}\text{X}\beta |{|}_{2}^{2}+\lambda \left(1-\alpha \right)\underset{G\in G}{\sum }||\beta_{G}|{|}_{2}+\lambda \alpha ||\beta_{G}|{|}_{1} \end{array}$$

Here λ is the strength of regularization, and α is the comparative strength of the *L*^1^ and *L*^2^ regularization, with indicating how much sparsity at the SNP level is desired compared to the sparsity at the group level. From a Bayesian perspective, one can think of it as adding a regularizing prior to **β** of the form:

(12) $$\begin{array}{l} \log p\left(\beta \right)\propto \left(1-\alpha \right)\underset{G\in G}{\sum }||\beta_{G}|{|}_{2}+\alpha ||\beta_{G}|{|}_{1} \end{array}$$

#### 2.1.4. Phenotype Prediction

With estimated **β** and δ, phenotype prediction follows from a straight-forward MLE using Equation (1). Suppose there are other samples with genotype ***X***′ and unknown phenotype ***y***′, then

(13) $$\begin{array}{l} LL\left({\text{y}}^{\prime }\right)\propto {\left(\left[\begin{array}{c} {\text{y}}^{\prime }\\ \text{y} \end{array}\right]-\left[\begin{array}{c} {\text{X}}^{\prime }\\ \text{X} \end{array}\right]\hat{\beta }\right)}^{T}{\left(\text{K}+\hat{\delta }\text{I}\right)}^{-1}\left(\left[\begin{array}{c} {\text{y}}^{\prime }\\ \text{y} \end{array}\right]-\left[\begin{array}{c} {\text{X}}^{\prime }\\ \text{X} \end{array}\right]\hat{\beta }\right) \end{array}$$

Here $\text{K}=\left[\begin{array}{cc} {\text{K}}_{{\text{X}}^{\prime }{\text{X}}^{\prime }} & {\text{K}}_{{\text{X}}^{\prime }\text{X}}\\ {\text{K}}_{{\text{X}}^{\prime }\text{X}}^{T} & {\text{K}}_{\text{X}\text{X}} \end{array}\right]$ So, by linear algebra, the MLE estimate for **y**′ is

(14) $$\begin{array}{l} \hat{y^{\prime }}=X^{\prime }\hat{\beta }+K_{X^{\prime }X}{\left(K_{XX}+\hat{\delta }I\right)}^{-1}\left(y-X\hat{\beta }\right) \end{array}$$

We can summarize the SGL-LMM significant SNPs selection in the following algorithm:

**Algorithm 1:** Parameter estimate for LMM with SGL regularization

**Data**: Genotype **X**, Phenotype **y**, α, λ
**Result**: ${\hat{\beta }}_{reg}$
Calculate ***K*** by selected genetic markers, orthogonal decompose it into ***U*** and ***S*** Estimate δ using Equation (6); Use the δ and ***S*** from above to evaluate ***S***_δ_; Calculate ${\hat{\beta }}_{reg}$ using Equation (11).

#### 2.1.5. Complexity Analysis

Let *n* be the number of samples and *s* be the number of SNPs. When training the null model, the complexity is *O*(*n*^3^) which is from the computation of eigenvalues and eigenvectors. This is reasonable when *n* is about 10k but for higher *n* one can improve on the time complexity by only taking into account the dominant eigenevalues. The proximal gradient step has a complexity of about *O*(*ns*), and since *n* is usually much less than *s*, one can see it as more or less *O*(*s*). The prediction step has a complexity of *O*(*nn*′*s*), where *n*′ is the size of the testing set. From the complexity analysis, we can see that SGL-LMM is scalable for the genome-wide association analysis. But when analysing with a huge genome such as the human genome, we recommend to analysis each chromosome individually or doing a 2nd step based on suggested loci from GWAS.

### 2.2. Model Selection

When solving the Equation (11), we employ SGL R package. Instead of doing a two dimensional grid search of λ and α to determine the optimal parameters, the package fix the mixing parameter α and compute solutions for a path with many λ values. The path begins with *lambda* sufficiently large to set $\hat{\beta }=0$ and let *lambda* decrease until the result is close to unregularized. Taking advantage of this mechanism, we carry out feature selection using LMM-SGL through the following steps:

(1) Finding the λ that optimizes phenotype prediction accuracy

In order to find the best λ for phenotype prediction, we first fitted the sparse group lasso model with the whole dataset to find a λ path. We then used 5-fold cross validation to find the appropriate λ, which maximize the average explained variance on the test dataset.

(2) Stability selection

To evaluate the significance of individual SNPs, we carry out stability selection (Meinshausen and Bühlmann, 2010). To obtain a more accurate ranking of SNPs, after the optimal λ was selected in the step above, we chose another 9 λs from the larger λs in the λ path evenly spaced. This group of λs were used in each stability selection process to rank the features by the order of inclusion into the model. We drew randomly no more than 50% of the samples as proposed in the original artical 100 times. We selected all SNPs that were found in ≥ 50% of all results. Significance estimate can be deduced from the selection frequency of individual SNPs.

**Figure 2:**
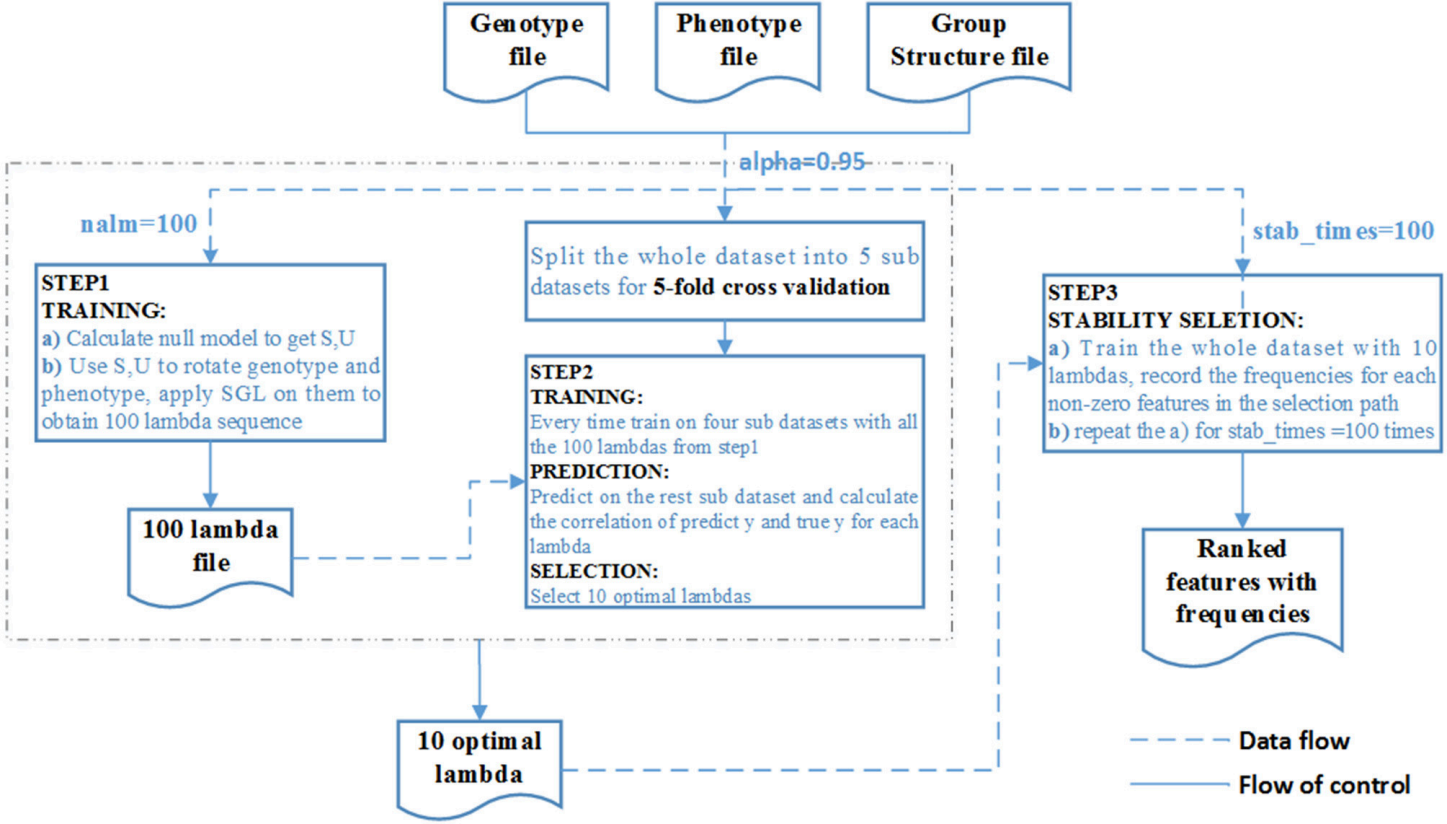
Flow chart of the SGL-LMM method. The dotted line shows the data flow, and the solid line shows the flow of control.

We summarize the process as the algorithm below and the overall pipeline of SGL-LMM method as Figure 2:

**Algorithm 2:** Feature selection using SGL-LMM

**Data**: *Genotype*, *Phenotype*, *groupstructure*, α, *nlam*_*times*
**Result**: List of features and their importance measured by frequencies
For a decreasing sequence of *nlam*_*times* different λs, use 5-fold validation to measure the performance of the result of Algorithm 1, pick the optimal λ; Pick another 9 λs larger than the optimal, evenly spaced in the λ-path used above, label them λ_*i*_, *i* = 1, 2, …10; Sample the data set 100 times, use all 10 λ_*i*_, estimate β using Algorithm 1. Output the features with non-zero coefficients in more than half of the estimates $\hat{\beta }$ and their frequencies

### 2.3. Simulation Study

To evaluate the accuracy of SGL-LMM and pervious methods for association mapping, we considered a semi-empirical example based on the genotypic and phenotypic data for up to 1307 world-wide accessions of *Arabidopsis thaliana* from Atwell et al. (2010). The data can be downloaded from https://github.com/Gregor-Mendel-Institute/atpolydb. Based on the quality control provided by GWAS, we excluded a SNP if its Minor Allele Frequency (MAF) was < 0.05, if its missing rate was >0.05 of the population, or its allele frequencies were not in Hardy-Weinberg equilibrium (*P* < 0.0001). After filtering, there were 200155 SNPs left.

To simulate the effect of population structure, we used the real phenotypic leaf number at flowering time (LN,16°C,16 h daylight) which is available for 177 plants of the 1307 plants of *A.thaliana*. Univariate analyses showed that the phenotype had an excess of associations when population structure was not taken into account (Atwell et al., 2010). After correction for population effect, the *p*-values are approximately uniformly distributed, Which means this phenotype is totally subjected to population structure. Hence, we use this phenotype to simulate the confounding effect. First, to determine the fraction δ of genetic and residual variance, we fit a random effects model to LN, which we subsequently used to predict the population structure for the remaining 1,130 plants. We run the random effect model multiple times, and choose the final dataset which the difference of genetic variance parameter between real and synthetic data are less than 0.0001. In addition to this empirical background, we added simulated association with different effect sizes and a range of complexities of genetic models.

We then simulated the phenotype as follows:

(15) $$\begin{array}{l} \text{y}=\sigma_{sig}{\text{y}}_{sig}+\left(1-\sigma_{sig}\right)\left[\sigma_{pop}{\text{y}}_{pop}+\left(1-\sigma_{pop}\right)\varphi \right] \end{array}$$

where ${\text{y}}_{sig}=X^{k}\beta$, ***X***^***k***^ is the genotype data for the k causal SNPs. By introducing the group structure, we consider a case with *N*_*g*_ = 200 genes(groups) on the chromsome1 which covered 2000 SNPs, we set *m* groups to be active. We vary the sparsity level of the active groups to get the total active SNPs to be k. β~N(0,I) and φ~N(0,I). During the simulation, we maintained the original LD structure in each gene.

The initial setting used for simulation were 3 active groups each containing 5 effective SNP (*k* = 15 and *m* = 3). To investigate the influence of the confounding effect strength and the overall noise, we considered varied σ_*pop*_ ∈ {0.5, 0.7, 0.9} and σ_*sig*_ ∈ {0.1, 0.2, 0.3, 0.4, 0.5}. For each combination of σ_*pop*_ and σ_*sig*_, we generate 10 datasets, resulting in 120 datasets in total for the 12 combinations.

### 2.4. Application With *Arabidopsis thaliana* Data

To assess the capacity of SGL-LMM to deal with real association mapping of quantitative phenotypes, we investigated the susceptibility of a set of SNPs that belong to genes of several flowering phenotypes in A. thaliana. We used the same dataset as in the simulation study. From the 107 phenotypes, we chose 10 flowering time phenotypes (Table S1).

To verify our method, we constructed our dataset in the following ways:

1. We obtained gene information from the A. thaliana annotation file. For each gene, 10kb of buffer region was added both upstream and downstream of the defined gene location. All SNPs between the regions were considered.
2. From chromosome 1 to chromosome 5, we chose the top 1,000 largest genes to form a genotype data file. There were a total 49,962 SNPs in the 1,000 genes.
3. According to the most promising association listed in Atwell's paper, we chose 19 genes that were related strongly to flowering time and added them to the genotype. The 19 genes consisted of 367 SNPs, so that the final genotype file had 50,329 SNPs (Table S2).
4. For each phenotype, a corresponding kinship matrix was generated in the same way as described in the simulation study.

## 3. Results

### 3.1. Existing Methods

To compare our SGL-LMM method with existing techniques, we considered standard regularization methods that included Lasso and SGL, which model all SNPs simultaneously without correcting for population structure. Also, we combined LMM with different regularization strategies (e.g., Lasso-LMM was listed as a comparison). All the methods that were related to regularization were fit in identical ways (see section 2.2).

### 3.2. Performance Measurements

In this paper, all the models output a ranking list of SNPs with their frequencies of being chosen; true significant markers were rare and accounted for only 15 out of 1,993 in our simulation datasets. Hence, we treated this as a binary classification problem with an imbalanced dataset where we assigned association markers as label 1 and background markers as label 0. The frequency of each marker was treated as the predicted probability for label 1.

The ROC (Receive operating characteristic) curve and the PR (Precision-Recall) curve are commonly used to evaluate performance of classification models. The ROC curve is created by plotting the Sensitivity against the Specificity while varying the threshold settings:

$$\begin{array}{l} sensitivity\left(TruePositiveRate,TPR\right)=\frac{TP}{TP+FN} \end{array}$$

$$\begin{array}{l} specificity\left(FalsePositiveRate,FPR\right)=\frac{TN}{TN+FP} \end{array}$$

The PR curve is created by plotting the Precision against the Recall at various threshold settings:

$$\begin{array}{l} precision=\frac{TP}{TP+FP} \end{array}$$

$$\begin{array}{l} recall=\frac{TP}{TP+FN} \end{array}$$

where TP=TruePositve, TN=TrueNegative, FP=FalsePositive, and FN=FalseNegative.

In our imbalanced setting, the ROC curve was not a good visual illustration, because the False Positive Rate did not drop drastically when the True Negative was huge. Whereas, the PR curve was highly sensitive to False Positive and was not impacted by a large True Negative denominator. Hence, we chose the PR curve to evaluate the performance for all the methods, and we used the average AUC (Area Under Curve) of the PR curve to explore the impact of various simulation settings.

### 3.3. Results of the Simulation Study

#### 3.3.1. *SGL-LMM Ranks Causal SNPs Higher Than Alternative Methods*

**Figure 3:**
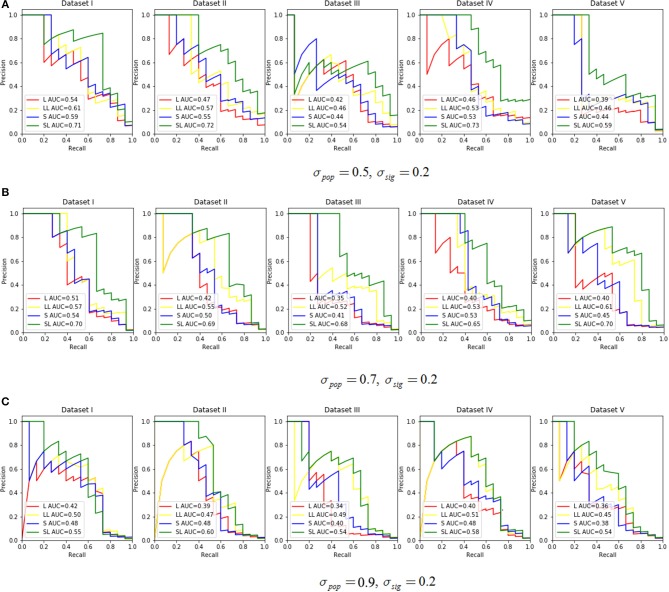
The Precision Recall curve by varying the σ_*pop*_ ∈ {0.5, 0.7, 0.9} and set σ_*sig*_ = 0.2. Each parameter combination had five datasets. The legend in each subplot shows the area under the curve (AUC) for each method. L represents Lasso only, LL means Lasso-LMM, S is SGL only, and SL means SGL-LMM. **(A)** Shows the PR curve under σ_*pop*_ = 0.5, σ_*sig*_ = 0.2, **(B)** for σ_*pop*_ = 0.7,σ_*sig*_ = 0.2, and **(C)** for σ_*pop*_ = 0.9,σ_*sig*_ = 0.2.

We assessed the performance in recovering causal SNPs with a true simulated association. PR curves were constructed while varying σ_*pop*_ in {0.5, 0.7, 0.9} with σ_*sig*_ set at 0.2 (Figure 3). Notice that a larger AUC score indicated better performance. For this experiment, we chose effective SNPs from three of the 200 groups, while taking sparsity into account, and we set the ratio α of L1 and L2 penalty in SGL-LMM to be 0.95. The two methods that incorporated LMM for population correction performed better than those without, and SGL-LMM was the best model (Figure 3). For most sets of parameters, SGL-LMM outperformed Lasso-LMM in AUC by about 10%.

**Figure 4:**
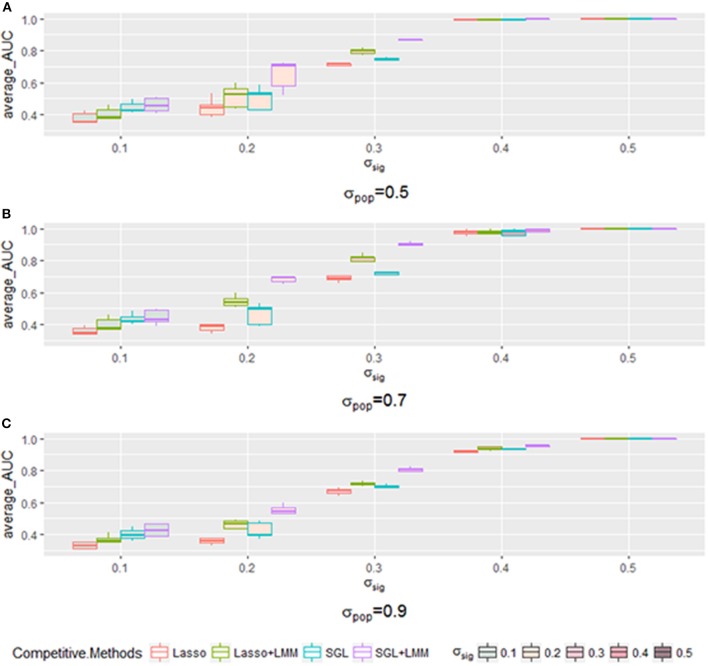
The boxplot of a sample of five points for each method with a specific when varying the σ_*pop*_. Each method has a different color frame, and each that is filled with a different color is shown in the legend. **(A)** for σ_*pop*_ = 0.5, **(B)** for σ_*pop*_ = 0.7, and **(C)** for σ_*pop*_ = 0.9.

Next, we explored the impact of various simulated setting. As mentioned in section 3.2, the area under the Precision-Recall curve is a summary performance measurement to assess different methods. The AUC under the PR curve is shown as a function of an increasing ratio between true genetic marker signals compared with confounding and noise (Figure 4). The performance of all methods improved when σ_*sig*_ became larger, and the *AUC* = 1 at σ_*sig*_ = 0.5 for all methods. Among them, SGL-LMM was the best. We also notice that when σ_*sig*_ = 0.1, only SGL was more accurate than Lasso-LMM in the majority of datasets. SGL and Lasso-LMM performed similarly (Figure 3). One possible explanation is that when the variation explained by causal SNPs was relatively small, noise dominated the results. Under this scenario, eliminating false positives caused by population structure did not improve the performance of the models significantly. However, imposing group structure seems to be useful in generating accurate results.

**Figure 5:**
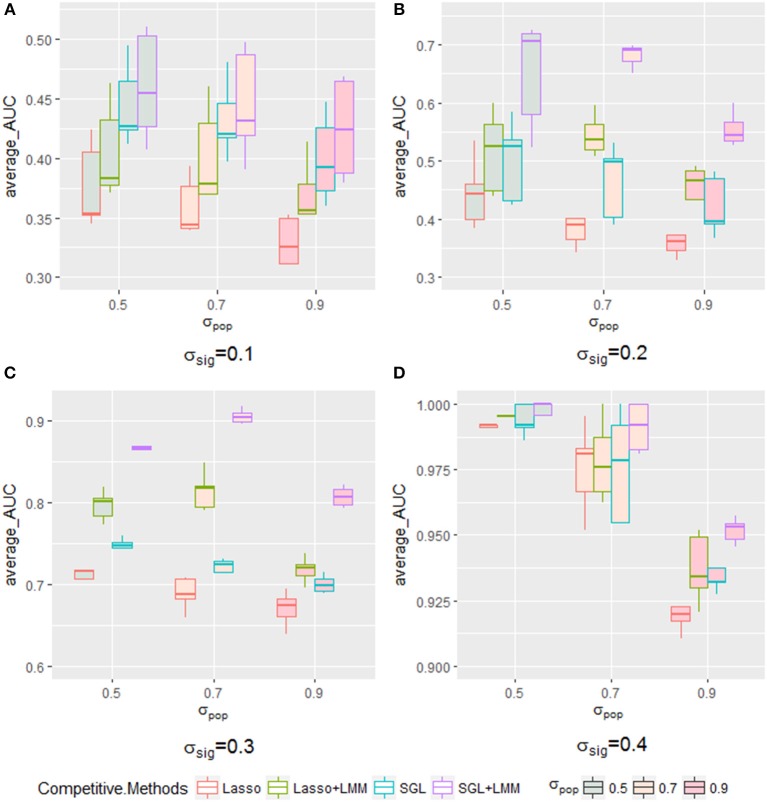
The boxplot of a sample of five points for each method with a specific σ_*sig*_ when varying the σ_*pop*_. Each method with a different color frame and each σ_*sig*_ filled with a different color are shown in the legend. **(A)** for σ_*sig*_ = 0.1, **(B)** for σ_*sig*_ = 0.2, **(C)** for σ_*sig*_ = 0.3, and **(D)** σ_*sig*_ = 0.4.

The AUC under the PR curve is shown as a function of an increasing ratio of population structure and independent random noise with a specific σ_*sig*_ and, as expected, strong confounding was harmful to performance, because the AUC of all methods decreased when the confounding ratio increased. Again, SGL-LMM was superior to its counterparts. However, when σ_*sig*_ = 0.3, the performance of methods with the population correction exhibited an upper trend when σ_*pop*_ varied from 0.5 to 0.7 (Figure 5C). The performance of δ_*sig*_ to be 0.1,0.2 and 0.4 can be found in Figures 5A,B,D. This effect indicated that with a medium signal to noise ratio, it was advantageous to include a genetic covariance matrix K that accounted for confounding that was caused by population structure. SGL-LMM performed better than alternative methods for the entire range of considered settings. The benefits of population correction and inclusion of group structure in SGL-LMM were most pronounced in the scenario with strong confounding.

### 3.4. Application With *Arabidopsis thaliana* Data

Having shown the accuracy of SGL-LMM in recovering the association SNPs in the simulation study, we can demonstrate that the SGL-LMM models association mapping in the A. thaliana dataset better than other models. For this experiment based on real data, we compared the performance of SGL-LMM and Lasso-LMM in predicting phenotype and in selecting predictive SNPs. For the ratio α between L1 and L2 penalty, we considered eight possible values {0.95, 0.85, 075, 0.65, 0.55, 0.45, 0.35, 0.25}; we picked the one that resulted in the largest correlation coefficient between the predicted and the real phenotype for subsequent stability selection. Because it is a verification experiment, we did not cover all genes in the experimental design. It may be the case that few, or even none, of the related genes in the selected phenotypes were covered in our genotype file. As a consequence, when setting the threshold for stability selection to be 50%, few SNPs are chosen by Lasso-LMM, and usually no more than 20 SNPs are chosen by SGL-LMM. Hence, we chose to rank the SNPs by their frequency of being chosen in both approaches and to investigate the first 100 SNPs. We summarized the genes to which these 100 SNPs belonged and the number of these genes in the candidate gene list (Table 1).

**Table 1:** Summary of associations found in SGL-LMM and Lasso-LMM in real data application.

**Phenotype**	**Method (lambda)**	**Correlation**	**Number of genes covered by top 100 SNPs**	**Number of genes in the 19 selected genes**
FT10	Lasso+LMM (1)	0.100938	90	4
	SGL+LMM (0.35)	0.231566	14	**10**
	SGL+LMM (0.85)	0.233074	36	**12**
FT16	Lasso+LMM(1)	0.184048	78	5
	SGL+LMM (0.95)	0.225247	61	**8**
FT22	Lasso+LMM(1)	0.228702	87	6
	SGL+LMM (0.85)	0.233883	31	**10**
LD	Lasso+LMM (1)	0.186646	85	7
	SGL+LMM (0.95)	0.278401	63	**9**
LDV	Lasso+LMM (1)	0.118177	80	6
	SGL+LMM (0.95)	0.168179	61	**7**
SD	Lasso+LMM (1)	0.267138	82	10
	SGL+LMM (0.95)	0.294031	53	10
SDV	Lasso+LMM (1)	0.050816	94	4
	SGL+LMM (0.25)	0.063342	14	4
LN10	Lasso+LMM (1)	0.053226	90	1
	SGL+LMM (0.25)	0.062286	12	0
LN16	Lasso+LMM (1)	0.040451	92	0
	SGL+LMM (0.85)	0.061766	45	0
LN22	Lasso+LMM (1)	0.062493	81	1
	SGL+LMM (0.45)	0.066171	13	1

*We report the correlation between the predicted phenotype and the real phenotype in the column titled “correlation.”. A bold entry indicates that the method located more true positives than its competitor*.

SGL-LMM had the following two advantages (Table 1):

#### 3.4.1. *SGL-LMM Had Higher Prediction Accuracy*

For most of the 10 phenotypes, correlation coefficients between the predicted and the true phenotypes were higher using SGL-LMM than those obtained with Lasso-LMM by >10%; for FT10, the predictions by SGL-LMM had a correlation coefficient 100% higher than that obtained by Lasso-LMM. Therefore, incorporating prior knowledge of genetic structure significantly improved the accuracy of models of quantitative phenotypes.

#### 3.4.2. *SGL-LMM Selected Fewer Genes, and It Tended to Find More Genes That Were Known to be Functional*

Compared with Lasso-LMM, associations that were located by SGL-LMM were more enriched to known candidate genes (Table 1). It linked more candidate genes in five phenotypes, and it linked the same number of candidate genes in the phenotypes SD and SDV. However, SGL-LMM linked many fewer genes compared with Lasso-LMM, which was consistent with our assumption that phenotypes should be related to a few SNPs in a few genes. Hence, adding group information into SGL-LMM made the results more interpretable and more meaningful biologically. The remaining three phenotypes that were related to leaf numbers seemed to be largely unrelated to the 19 candidate genes and to the randomly selected background genes and, therefore, both methods performed badly.

## 4. Discussion

Quantitative traits are important in medicine, agriculture, and evolution, but the association mapping studies of these traits are insufficient. In this paper, we have proposed a sparse group lasso, multi-marker mixed model (SGL-LMM) to identify genetic associations in quantitative traits with the presence of confounding influences, such as population structure. The approach benefits from the attractive properties of linear mixed models that allow for elegant correction of confounding effects and those of group-based, multi-marker models that not only consider the joint effects of sets of genetic markers rather than one single locus at a time, but that also incorporate biological group information as prior knowledge. As a consequence, SGL-LMM was able to better predict the phenotype and to identify true genetic associations, even in challenging scenarios with complex underlying genetic models, weak effects of individual markers, or presence of strong confounding effects.

SGL-LMM is useful for genome-wide association studies of complex quantitative phenotypes. In this paper, we have illustrated such practical use through a semi-empirical simulation study and retrospective analysis of *A. thaliana*. First, we found that imposing gene structure as group structure into the model improved both the prediction of phenotype from genotype and the selection of association SNPs, which suggested that incorporating prior biological knowledge into models led to a better fit to real genetic architectures. Second, the combination of a random effect model and a multivariate linear model is a way to reveal the true association of complex phenotypes, especially with a medium signal to noise ratio. It is widely accepted that parts of the unexplained portion of genetic variance can be due to a large number of loci that have a joint effect on the phenotype, but which lead to only a weak signal if considered independently. In addition, SGL-LMM yields much more biologically meaningful and interpretable associations, which suits the biological assumption that complex traits are only related to a few SNPs in a few genes. Our experiments on the flowering phenotype of *A. thaliana* showed that SGL-LMM linked many more candidate genes, but this was true only in a smaller gene set compared with the Lasso-LMM method.

The SGL-LMM included both GL-LMM (group lasso with linear mixed model) and Lasso-LMM as special cases by varying the ratio between the L1 and L2 norms. The sparsity within groups and group-wise sparsity influenced the performance of SGL-LMM. Small groups did not benefit from the within-group sparsity that led the method act as group lasso with LMM. In practical use, we recommend doing imputation first, which can ensure a moderate size for each group. The SGL-LMM can be made even more powerful by adding a strategy to deal with overlapping groups, which has been shown to be feasible by Jacob et al. (2009). Assessing the statistical significance of association results of SGL-LMM remains a challenge for future research. In summary, SGL-LMM is a useful addition to the current toolbox of computational models for unraveling associations of quantitative traits.

## Author Contributions

YG, AK, MG, and XL conceived and designed the project. YG and CW derived the formula of the method. YG implemented the software, performed the experiment, analyzed data, and wrote the paper with CW and QZ. All authors read, edited, and approved the final version of the manuscript.

### Conflict of Interest Statement

The authors declare that the research was conducted in the absence of any commercial or financial relationships that could be construed as a potential conflict of interest. The reviewer YWC declared a past co-authorship with one of the authors QZ to the handling editor.
