# Supplementary material for: Combining Sparse Group Lasso and Linear Mixed Model Improves Power to Detect Genetic Variants Underlying Quantitative Traits
Source: Front Genet. 2019 Apr 10;10:271. doi: 10.3389/fgene.2019.00271 (PMC6469383; doi:10.3389/fgene.2019.00271)
Supplement: Supplementary file 1 [file Data_Sheet_1.PDF]

Supplementary Material

1 SUPPLEMENTARY TABLES

[1] The abbreviation of gene names

Table S1: Description of the 10 flowering related phenotypes in *A. thaliana* used in real data application

Phenotype	Accessions	Phenotype Description	Growths conditions	Phenotype scoring
LD	167	Days to flowering time(FT)	18°C 16hr daylight	Number of days following stratification to opening of
LDV	168	under Long Day (LD) and	18°C 16 hr daylight, vernalized(5wks 4)	the first flower. The experiment was stopped at 200d,
SD	162	Short Days (SD) +/- vernalization	18°C 8hr daylight	and accessions that had not flowered at the point
SDV	159		18°C 8hr daylight, vernalized (5wks 4)	were assigned a value of 200.
FT10	194	Flowering time (FT) and leaf	10°C 16hr daylight	Plants were checked bi-weekly for presence of first
FT16	193	number at flowering time (LN)	16°C 17hr daylight	buds, and the average flowering time and average leaf
FT22	193		22°C 18hr daylight	number of four plants of the same accession at each
LN10	177		10°C 19hr daylight	temperature were collected.
LN16	176		16°C 20hr daylight	
LN22	176		22°C 21hr daylight	

Table S2. Description of the candidate genes for flowering related phenotypes, chosen from Atwell et al (ref.1) and flowering time pathway (https://www.wikipathways.org/index.php/Pathway:WP2312)

Gene Name	Abbr[1]	Chr	Number of SNPs	Database Reference
Related to AB13/VP1	RAV1	1	24	AT1G13260(TAIR)
SPA1-related 4	SPA4	1	15	AT1G53090(TAIR)
Squamosa promoter binding protein-like 4	SPL4	1	11	AT1G53160(TAIR)
Flowering locus T	FT	1	31	AT1G65480(TAIR)
Hua enhancer 2	HEN2	2	21	AT2G06990(TAIR)
Short vegetative phase	SVP	2	8	AT2G22540(TAIR)
Similar to reduced vernalization response	VRN1	3	17	AT3G18990(TAIR)
Cryptic precocious	CCT	4	18	AT4G00450(TAIR)
Frigida	FRI	4	19	AT4G00650(TAIR)
Enhancer of TRY and CPC3	CPL3	4	30	AT4G01060(TAIR)
Dwarf in light 2	DFL2	4	18	AT4G03400(TAIR)
SPA 1-related 2	SPA2	4	25	AT4G11110(TAIR)
A.thaliana homeobox	ATH1	4	13	AT4G32980(TAIR)
Gibberellin 20 - oxidase 3	YAP169	5	9	AT5G07200(TAIR)
Flowering locus C	FLC	5	16	AT5G10140(TAIR)
Enhancer of AG-4 2	HUA2	5	18	AT5G23150(TAIR)
Flowering promoting factor 1	FPF1	5	19	AT5G24860(TAIR)
Delay of germination 1	DOG1	5	32	AT5G45830(TAIR)
Vernalization insensitive 3	VIN3	5	23	AT5G57380(TAIR)
